# Monthly Summary

**Published:** 1876-05

**Authors:** 


### MONTHLY SUMMARY.

*Chloral as an Anœsthetic.*—M. Bouchut stated, at the Congress of Brussels, that he obtains anæsthesia in children with from forty-five to sixty grains of chloral at one dose. It is especially in very severe cases of chorea that most benefit has been obtained. The dose just mentioned was continued for a month or six weeks, so that the same little patient absorbed, during that time, no less than seven or eight ounces of chloral. When from forty-five to sixty grains are given at one dose, the child soon goes to sleep, and anasthesia is complete in about one hour. If then an abscess is to be opened, the incision is made, the child being asleep. The latter may just sigh or groan, but falls asleep again ; and when it awakes, three or four hours afterwards, there is no knowledge of what has been done.

When teeth are to be extracted, they manage in this way : At eight o'clock a. m. the nurse gives forty-five or sixty grains, according to age, at one dose, and the child falls asleep in twenty minutes. At nine o'clock the dentist comes, and extracts the carious tooth ; or even two may be removed. The child groans and moves about without waking, and soon becomes again motionless. After about four hours the child awakes, and knows nothing of the operation.

With children, then, chloral seems an excellent anæsthetic in the doses mentioned above ; but it should be noted that no such results can be obtained with adults.—*Ibid.*—*Nashville Journal of Medicine and Surgery.*

---

*Painless Opening of Abscesses.*—Dr. Bergonzini uses a solution of two parts of carbolic acid with one part of glycerine, and leaves the mixture in contact with the skin for three or five minutes. Redness or swelling never occurs, except the skin had been previously inflamed, or the liquid allowed to remain on too long. This anæsthetic agent might, according to the author, be used in autoplastic operations, and he intends to try it in neuralgia where the seat of the pain is very superficial.—*London Lancet.*

*The Blue Line in Lead Poisoning.*—In order to distinguish the gingival line caused by lead from other discolorations on the gums which may resemble it, M. Cras, excises the edge of the gum, and examines it under the microscope. He finds that the lead-line does not result from a tattooing of the gums by some metallic particles having penetrated into the epithelial cells, or more deeply. It is due to transformation of a soluble salt of lead into a sulphide precipitated in the capillaries by the retarded circulation in the gums, owing to the vicinity of the putrid detritus surrounding the neck of the teeth. It is then, an injection of the capillaries by the sulphide of lead. M. Cras supposes that analogous vascular obliterations in the intestines are, perhaps, the cause of saturnine colic.—*Arch de Med. Nav.*

---

*Resection of a Portion of the Lower Jaw.*—Through the kindness of my friend, Dr. Carpenter, of New Lancaster, I was permitted to examine a boy, aged about twelve years, residing in a distant part of the country. A history of the case, as given by the uncle of the boy, corroborated by a careful personal inspection, revealed the existence of a large tumor attached to and springing from the right side of the lower jaw. It first appeared after the removal of a decayed tooth. and in a short time the adjacent teeth were loosened and displaced, when the tumor, in a few months, assumed the proportions of a large sized pullet's egg. When I saw him the tumor was intruding upon the space occupied by the tongue, interfering materially with the processes of mastication and deglutition, and emitting a very offensive discharge.

My diagnosis was that the tumor was of the nature of an aggravated form of epulis, and I announced as my opinion that nothing short of an entire removal of the growth, and the parts from which it sprang, would be of any avail. Accordingly on the second day of January, 1874, assisted by Drs. Carpenter, Hoover, Johnson and others, I proceeded to operate. We were satisfied that, to get beyond the diseased mass, I must cut through the jaw in the space occupied by the outer lateral incisor of the left side. Having, therefore, removed all the teeth on the right side and up to the point indicated, and the patient carefully chloroformed by Dr. Hoover, I proceeded to cut through the soft parts, cutting downward from the left corner of the mouth, leaving the lip intact, to the base of the jaw, extending the incision along the same to a point beyond the angle. Turning over the soft parts, having checked the bleeding by the usual means, I severed the jaw at the point already named, with the bone forceps and a Hey's saw ; and then dissecting the diseased from the healthy tissues, I divided the jaw at the angle.

The hemorrhage, which was not excessive, was readily controlled with a few ligatures and the application of styptics. This done, the wound, having been properly cleansed, was closed, and the parts held *in situ* by the necessary number of twisted and interrupted sutures, and the dressing completed with the usual compress and four-tailed bandage.

The patient was left in charge of Dr Carpenter, to whom we are indebted largely for the results, which are satisfactory to a degree beyond the most sanguine expectations. The boy recovered from the effects of the operation in a very short time. Soon the space occupied by the portion of jaw removed began to fill up with cartilaginous substance, which after a while began to harden and become osseous; and now, after the lapse of only two years, his jaw is, as it was before the operation, bony in all its parts, and the contour of the face preserved, with no marks of the ravages of the saw, forceps, and scalpel, except in the skin.

Believing that the results herein referred to will be viewed with interest by the profession, I venture to give them for what they are worth.—*Med. & Surg. Reporter.*

---

*Topical Application in Painful Dentition.*—

| | |
|---|---|
| ℞ Syrup of Tamarinds - - . | 2½ drachms. |
| Infusion of Saffron, - - - - | 2 scruples. |
| Honey, - - - - - | 2½ drachms. |
| Tinct. (essence) of vanilla, - - | 4 drops. |

Rub gently over the gums with the finger or rag. An application of a similar character is the following:

| | |
|---|---|
| ℞ Saffron (powdered) - - | 4 to 6 grains. |
| Honey, - - - - - | 2 to 3 drams. |

Glycerine may be substituted for the honey.—*Med. Brief.*

---

*Milk as a Vehicle of Contagion.*—In a recent pamphlet by Mr. A. H. Smee, an English sanitarian, the author reaches the conclusion that there is undoubted evidence to show that milk can be the vehicle of contagion:

By direct communication of the contagion, either by the water used for the purposes of adulteration, or by the vessels in which it is stored being cleansed with impure water.

By the absorption of the contagion by the exposure of milk to deleterious gases.

That in extreme instances power to communicate disease is produced in the milk itself, probably from an altered secretion of diseased animals.—*Med. & Surg. Reporter.*

*Avoidance of Phosphorous Poisoning.*—At the Brussels Congress there was presented the following report of M. Crocq, "On the Sanitary Measures in Workshops where Phosphorus is manipulated." The following conclusions were adopted: The section of public medicine expresses the wish, 1. That the use of red amorphous phosphorus be substituted for that of ordinary phosphorous in all match-factories. 2. Until the universal adoption of this radical measure, it recommends, in the actual conditions of manufacturing, the following measures, which are designed to prevent general toxic accidents, and more especially maxillary necrosis; installations of the manufacturing in sufficiently spacious rooms; powerful ventilation promoted by the aid of tubes, beginning at the ground and terminating in a drawing chimney; constant attention to cleanliness; together with these physical means, use as a chemical antidote, the spirits of turpentine. 3. Local accidents may be averted by astringent gargarisms, and above all, by the obligation imposed upon manufactures to admit none into their workshops who, by oral examination, present dental lesions, such as penetrating decay, or any other affection of a nature to favor the deleterious action of phosphoric vapors. 4. Children not to be employed in workshops where phosphorus is used. 5. When the authorities allow the establishment of manufactories where that substance is used, they must impose these conditions and see that they are observed, for the interests of workmen as well as of manufacturers, who are criminally responsible for accidents resulting from their carelessness or neglect.—*Med. & Surg. Reporter.*

---

*Borax as an Antiseptic.*—The antiseptic properties of borate of soda are being discussed in France, in connection with a proposition to preserve meats and milk by its agency.—*Ibid.*

---

*Necrosis of the Lower Jaw.*—Dr. A. C. Post presented a specimen of dead bone removed, June 5th, from the lower jaw of a child nine years old. The disease of the jaw had presented itself without any obvious cause, The mother of the child regarded it as the result of toothache, and it was probable that inflammation of the periosteum extended from the affected tooth. A portion of dead bone was found projecting through the gums, and was removed with a pair of strong forceps. Several forming teeth were involved in the disease. As is the case ordinarily when necrosis of the lower jaw occurs, there was a large amount of involucrum formed. When the disease affects the upper jaw, as a rule, scarcely any involcrum is formed.—*Med. Record.*

*The Discovery of Anœsthesia.*—" In Mount Auburn cemetery, near Boston, there stands to-day a monument erected by loving hands to the memory of Dr. Morton. On the four faces of it there are inscribed, in order, the legends. " Inventor and revealer of anæsthetic inhalation. Before whom, in all time, surgery was agony. By whom pain in surgery was averted and annulled. Since whom science has controlled pain" In the light of the above record, which I believe I have stated with accuracy, and I know without prejudice or favor, I simply ask, are these inscriptions true? Modify the first so that it shall read, " Inventor and revealer of the anæsthetic inhalation of sulphuric ether," and not the most rigid constructionist can take exception to the statement, for such honor is assuredly due him, and will remain to the end of time. But can we even then reconcile the remaining legends with the impartial record of history? I fear not, and I believe, rather than when the story of anæsthesia shall have been fully and finally written up, this verdict will be rendered in favor of the obscure dentist, whose memory has been neglected and whose name has been almost forgotten—*Dr. Horace Wells*, of Hartford, Connecticut. —Prof. E. S. Dunster, M. D., Ann Harbor, Michigan.—*Med. Record.*

---

*To Check Epistaxis.*—Dr. J. Gardner, writes to the *British Medical Journal* as follows :

" Last year, an old gentleman under my care, between seventy and eighty years of age, who had periodical attacks of bleeding from the nostrils, which had generally been stopped by plugging. was becoming exhausted from the loss of blood ; and the usual application not succeeding, I mixed equal parts of the tincture of sesquichloride of iron and water, about two drachms of each, and injected it up the nostrils with a glass syringe. This immediately stopped the bleeding, The bleeding, however, should not, I think, be checked too soon, as in most instances it is a provision of nature to relieve the vessels of the head. I have found, also, springing with a glass syringe of great benefit in throat affections, where there is difficulty in using a gargle, especially in children."

---

*Female Students.*—The number of young women who are studying medicine at St. Petersburg has considerably increased. The level of their attainments is also higher, and the reports of the professors are more favorably. The feminine students are 171 in number, of whom 23 are married women, 102 noble, 17 daughters of traders, 12 of ministers of religion, and 15 of families of the middle classes.

During the past session two ladies have been attending the lectures at the Philadelphia College of Pharmacy:—*Med. and Surg. Reporter.*

*Medicated Ice*—The possibility of using frozen antiseptics in medicine was recently pointed out by Mr. Edward Martin, in a letter to the *Lancet*, as follows—" Every practitioner has at times to face the difficulties of the scarlatinal throat in young children. It may sadly want topical medication; but how is he to apply it? Young children cannot gargle, and to attempt the brush or the spray often fills them with terror. In many cases neither sternness nor coaxing avails. Yet these little ones in every case will greedily suck bits of ice. This has long been my chief resource where I could not persuade the child to submit to the sulphurous-acid spray. Lately I have been trying an ice formed of a frozen solution of the acid (or some other antiseptic.) Though of course not so tasteless as pure ice, the flavor is so much lessened by the low temperature, and probably also through the parched tongue, very little appreciating any flavor whatever, that I find scarcely any complaint on that score from the little sufferers; they generally take to it very readily. The proces of making it is very simple; a large test-tube immersed in a mixture of pounded ice and salt is the only apparatus required, and a momentary dip of the tube in hot water enables one to turn out the cylinder of ice as the cook turns out her mould of jelly. I have tried the three following formulæ, all of which answer, though I think I prefer the first:

1. Sulphurous acid, half a drachm; water seven drachms and a half; mix and freeze.

2. Chlorate of potassium, one scruple; water, one ounce; dissolve and freeze.

3. Solution of chlorinated soda, half a drachm; water, one ounce; mix and freeze.

However, the form is of secondary importance, as each practitioner can construct his own. Boracic acid, salicylic acid, or any other harmless antiseptic with not too much taste, would, doubtless, be as useful as those indicated.—*Jour. of Materia Medica.*

---

*Extracting Teeth for Brain Disease.*—A correspondent writes: "What do you think of extracting the temporary teeth in a child two years of age, for brain trouble? It is practiced in my neighborhood, and by a 'knowing one.'" To which we answer—the practice is, we think, a relic of antiquity. We can, however, readily conceive of cases in which the brain of a child is threatened with disease, and in which the extraction of the teeth might do good by a diversion of excitement. The theory of its therapeutic action is probably the same that applies to chopping off the end of the tail of a sick dog.—*Pacific Med. & Surg. Journal.*

*The Plethysmograph.*—This is the name of an instrument invented by Dr. Mosso, of Turin, to measure the action of the blood vessels in the human body. The apparatus is described as a sort of muff; made of vulcanized India-rubber, somewhat sfmilar to the apparatus employed by Junod for dry-cupping. This is filled with water, at the temperature of the body, and so arranged as completely to envelop the member to be experimented on. The water in the muff is made to communicate with a cupel, by means of two glass tubes, which are equally filled with water, suspended over another liquid of less density. As soon as the slightest motion occurs in the muscles or blood-vessels of the limb (the forearm, for instance,) the water rises or falls according to the degree of motion imparted to it. In other words, if the blood-vessels of the forearm be dilated, the water in the tube rises; if contracted, the water falls. A needle fixed to one of the tubes traces an interrupted line, which faithfully represents the succession of movements imparted to it through the liquid. M. Claude Bernard observed that the use of the apparatus may be of a more extended application than would appear at first sight. "It may serve to study and demonstrate some of the most important phenomena of the blood-vessels of the body; and it affords the means of solving questions of a more general character, appertaining to the physiology of the mind, of the action of the brain and of the conscience."—*Med. and Surg. Reporter.*

---

*For Burns.*—

| | | |
|---|---|---|
| ℞ | Glycerine, - - - | 5 ounces. |
| | White of Egg, - - - | 4 ounces. |
| | Tincture of Arnica, - - | 3 ounces |

Mix the Glycerine and white of egg intimately in a mortar, and then add gradually the Arnica. Apply freely on linen cloths night and mornfng, having previously washed the burn with warm Castile suds.—*Medical Brief.*

---

*Cosmoline.*—This preparation is one of the distillations from petroleum. It is semi-solid, with an unctuous feel, and might readily be mistaken for one of the thinner animal oils. It has the important property of never turning rancid under any conditions of heat or moisture It is in fact, strongly antiseptic, and hence is admirably adapted to many skin diseases, preventing pruritus, protecting from the air, and opposing morbid action. For burns, scalds, bites of insects, erythema, solare, and similar affections, it is an admirable application. We make a rule to keep a bottle of it constantly at hand.—*Med & Surg. Reporter.*

